# Adenovirus Co-Opts Neutrophilic Inflammation to Enhance Transduction of Epithelial Cells

**DOI:** 10.3390/v14010013

**Published:** 2021-12-22

**Authors:** James M. Readler, Meghan R. Burke, Priyanka Sharma, Katherine J. D. A. Excoffon, Abimbola O. Kolawole

**Affiliations:** Department of Biological Sciences, Wright State University, Dayton, OH 45435, USA; readler.3@wright.edu (J.M.R.); jenkins.243@wright.edu (M.R.B.); priyankasharma18@gmail.com (P.S.); katherine.excoffon@wright.edu (K.J.D.A.E.)

**Keywords:** adenovirus, entry, autophagy, neutrophil, human neutrophil elastase, MDCK epithelial cells

## Abstract

Human adenoviruses (HAdV) cause a variety of infections in human hosts, from self-limited upper respiratory tract infections in otherwise healthy people to fulminant pneumonia and death in immunocompromised patients. Many HAdV enter polarized epithelial cells by using the primary receptor, the Coxsackievirus and adenovirus receptor (CAR). Recently published data demonstrate that a potent neutrophil (PMN) chemoattractant, interleukin-8 (IL-8), stimulates airway epithelial cells to increase expression of the apical isoform of CAR (CAR^Ex8^), which results in increased epithelial HAdV type 5 (HAdV5) infection. However, the mechanism for PMN-enhanced epithelial HAdV5 transduction remains unclear. In this manuscript, the molecular mechanisms behind PMN mediated enhancement of epithelial HAdV5 transduction are characterized using an MDCK cell line that stably expresses human CAR^Ex8^ under a doxycycline inducible promoter (MDCK-CAR^Ex8^ cells). Contrary to our hypothesis, PMN exposure does not enhance HAdV5 entry by increasing CAR^Ex8^ expression nor through activation of non-specific epithelial endocytic pathways. Instead, PMN serine proteases are responsible for PMN-mediated enhancement of HAdV5 transduction in MDCK-CAR^Ex8^ cells. This is evidenced by reduced transduction upon inhibition of PMN serine proteases and increased transduction upon exposure to exogenous human neutrophil elastase (HNE). Furthermore, HNE exposure activates epithelial autophagic flux, which, even when triggered through other mechanisms, results in a similar enhancement of epithelial HAdV5 transduction. Inhibition of F-actin with cytochalasin D partially attenuates PMN mediated enhancement of HAdV transduction. Taken together, these findings suggest that HAdV5 can leverage innate immune responses to establish infections.

## 1. Introduction

Human adenovirus (HAdV) is a double stranded DNA, pathogenic virus with a wide tissue tropism [1]. It is a frequent cause of self-limited gastroenteritis and upper respiratory tract infections in otherwise healthy hosts, but can cause severe disease in immunocompromised individuals [2]. For example, as many as 7.2% of pediatric solid organ transplant patients test positive for HAdV infections and disease severity can range from chronic diarrhea to fulminant pneumonia, viremia, and sometimes death [3,4]. Currently, there are no specific anti-adenoviral therapeutics on the market, meaning that clinicians must rely on the use of antivirals developed against other viruses that have some activity against HAdV, such as the potentially nephrotoxic cidofovir [1,5]. Importantly, human adenovirus serotype 5 (HAdV5) has been thoroughly studied for decades and is currently being used as a vector system to treat a variety of diseases, including cancer, as oncolytic virotherapies, and the COVID-19 pandemic as novel vaccines [6,7,8]. Thus, enhancing the understanding of the molecular mechanisms that govern HAdV transduction of target cells holds the potential to reveal therapeutic targets for the treatment of HAdV infections and the facilitation of HAdV vector delivery.

There is accumulating evidence suggesting that HAdV has evolved to co-opt various innate immune mechanisms to facilitate the establishment of infection. Autophagy is a highly evolutionarily conserved process of regulated digestion of cellular components [9]. This process serves important roles for cell survival during nutrient starvation, turnover of malfunctioning organelles, and host defense against intracellular pathogens [10,11,12]. HAdV relies on a PPxY motif on its capsid to escape newly formed autophagosomes in host cells, thereby preventing eventual lysosomal degradation [13]. Interestingly, cells that are actively undergoing autophagic flux have been shown to be more susceptible to HAdV2 transduction through a mechanism that is believed to involve more efficient endosomal escape [14]. Most human HAdV enter epithelial cells using their primary receptor: the Coxsackievirus and adenovirus receptor (CAR) [15,16]. In a polarized epithelium, the majority of CAR localizes to the basolateral membrane where it is largely inaccessible to luminal HAdV. However, the eight exon-encoded apical isoform of CAR (CAR^Ex8^) is capable of mediating apical HAdV infection of polarized epithelia [17]. Additionally, exposure to innate proinflammatory cytokine interleukin-8 (IL-8) results in increased airway epithelial expression of CAR^Ex8^, which mediates increased HAdV5 epithelial transduction [18,19]. IL-8 is a potent neutrophil (PMN) chemoattractant, and incoming PMN are capable of binding CAR via the transmembrane protein, junction adhesion molecule-like (JAML) [20,21]. The upregulation of CAR^Ex8^ leads to enhanced PMN adhesion to the apical surface of polarized epithelia [18]. Furthermore, exposure to primary human PMN results in an enhancement of epithelial HAdV5 transduction through a previously unknown mechanism [18]. In this study, we set out to elucidate the mechanism behind PMN-mediated enhancement of HAdV5 transduction, and we show for the first time that the serine protease human neutrophil elastase (HNE) enhances HAdV5 transduction of an epithelial cell model through a mechanism that involves the activation of autophagic flux.

## 2. Materials and Methods

### 2.1. Ethics Statement

Primary human neutrophils were isolated from the peripheral blood of healthy human donors according to the guidelines set forth by the Institutional Review Board at Wright State University (IRB SC# 6092).

### 2.2. Cell Culture and Reagents

We used a Madin–Darby Canine Kidney (MDCK) model system that stably express CAR^Ex8^ under a doxycycline inducible promoter, which allowed us to recapitulate the IL-8 signaling effect on apical CAR^Ex8^ in a polarizable epithelium without having to treat cells with IL-8. The MDCK-CAR^Ex8^ cell line was produced using a Lenti-X-Tet-On hAdVanced inducible expression system according to the manufacturer’s instructions (Takara Bio USA, San Jose, CA, USA) and as previously described [18]. Cells were cultured in minimum essential media (MEM) containing 5% (*v*/*v*) tetracycline free FBS. MDCK-CAR^Ex8^ cells were seeded in 24-well plates (Thermo Fisher Scientific, Waltham, MA, USA) at a density of 2.4 × 10^4^ cells/well for 24 h. CAR^Ex8^ expression was then induced by incubating cells in complete media containing 50 ng/mL doxycycline for an additional 24 h. During polarization experiments, 2 × 10^6^ MDCK-CAR^Ex8^ cells were seeded on 30-mm transwell inserts with 0.4-μm pore size (MilliporeSigma, Burlington, MA, USA). Cells were polarized in transwell inserts until the surface layer became dry, and transepithelial resistance was >600 Ω·cm^2^ as measured using a Volt/Ohm Meter with STX2 “chopstick” electrode (World Precision Instruments, Sarasota, FL, USA) as previously described [22]. All drug treatments were performed in serum-free media or in Hanks Balanced Salt Solution (HBSS) during starvation experiments and PMN treatment. Cytochalasin D (Life Technologies, Carlsbad, CA, USA) was used at a concentration of 5 μM for 20 min at 37 °C. Bafilomycin A1 (Alfa Aesar, Haverhill, MA, USA) was used at a concentration of 100 nm for 4 h at 37 °C. 4-(2-aminoethyl)benzenesulfonyl fluoride hydrochloride (AEBSF) (Thermo Fisher Scientific, Waltham, MA, USA) was used at a concentration of 200 μM for 1 h at room temperature. Dynasore (Tocris Bioscience, Bristol, UK) was used at a concentration of 100 μM for 1 h. Amiloride (Tocris Bioscience, Bristol, UK) was used at a concentration of 5 mM for 1 h.

### 2.3. Adenovirus Transduction

The adenovirus used in these experiments was a replication-incompetent HAdV5 containing the LacZ reporter gene (HAdV-LacZ). It was purchased from the University of Iowa Viral Vector Core at a titer of 2 × 10^10^ pfu/mL. Viral inoculations were performed in serum free media for 1 h at 37 °C at an MOI of ~125. Unbound virus was then washed away with phosphate buffered saline (PBS), and cells were kept in complete media at 37 °C overnight. The following day, cells were lysed, and beta-galactosidase activity assay was performed using a Galacto-Light Plus Beta-galactosidae Reporter Gene Assay System (ThermoFisher Scientific, Waltham, MA, USA) according to the manufacturer’s instructions and as previously described [22].

### 2.4. PMN Isolation and Co-Culture

Primary human PMN were isolated from the peripheral blood of healthy human volunteers. Approximately 60 mL of whole blood was drawn into EDTA tubes by a licensed phlebotomist. PMN were separated using 6% dextran followed by centrifugation through a Ficoll gradient as previously described [18]. PMN were washed and stored in HBSS on ice until ready for use in experiments. All PMN were used for experiments within the first few hours after isolation and were never frozen for later use. Viable PMN were quantified using a hemocytometer in conjunction with Trypan Blue stain. The appropriate volume of PMN stock was then separated into a 15-mL conical tube and centrifuged 740× *g* for 4 min to pellet the PMN. PMN were reconstituted such that 2 × 10^6^ PMN were suspended in 50 μL of HBSS containing calcium and magnesium (HBSS +/+). This PMN suspension would be added to each well of 24-well plate of MDCK-CAR^Ex8^ cells containing 250 μL of serum free media and rocked gently. MDCK-CAR^Ex8^ cells and PMN co-cultures were then placed at 37 °C for 15 min. PMN were removed from MDCK-CAR^Ex8^ cells with three HBSS washes.

### 2.5. Western Blot

Cells were washed multiple times with PBS and then incubated in lysis buffer on ice for 10 min and then manually scraped using a cell lifter. Lysis buffer contained 50 mM Tris pH 7.4, 150 mM NaCl, 1% Triton X-100, as well as the protease inhibitors leupeptin (10 mg/mL), apoprotinin (10 mg/mL), pepstatin (10 mg/mL), phenyl-methylsulfonyl fluoride (1 mM), and AEBSF (5 mM). Lysates were then sonicated 3 pulses each and centrifuged at 17,000 RCF at 4 °C for 10 min. Protein was quantified using a Bio-Rad Protein Assay according to the manufacturer’s instructions. Lysates were mixed with 2× loading dye containing 4% (*w*/*v*) SDS, 20% (*v*/*v*) glycerol, 3.25% (*v*/*v*) 2 M Tris pH 6.8, 120 μM bromophenol blue, and 100 mM dithiothreitol. These lysate/loading dye mixtures were then heated at 65 °C for 10 min. Equal amounts of protein were loaded into 4–20% Mini-PROTEAN TGX Precast Protein Gels (Bio-Rad, Hercules, CA, USA) and blotted onto polyvinyl fluoride membranes using a Trans-Blot^®^ Turbo™ Transfer System (Bio-Rad, Hercules, CA, USA). Blots were stained with Ponceau S stain as a loading control and then cleaned with 0.1 N NaOH followed by multiple washes in DI water. Blots were then blocked in 5% BSA in Tris-buffered saline with 0.1% Tween 20 detergent (TBST) for 30 min at room temperature. Antibodies were diluted in blocking buffer and rocked on blots overnight at 4 °C. Anti-CAR^Ex8^ antibody was generated by Genescript (Piscataway, NJ, USA) as previously described and used at a concentration of 1:3000 [23]. Anti-p62 antibody was purchased from Abcam (Cambridge, UK) and used at a concentration of 1:3000. Anti-LC3BII antibody (1 mg/mL) was purchased form ThermoFisher Scientific (Waltham, MA, USA) and used at a concentration of 1:2000. All images were taken with an Amersham Imager 600 (General Electric, Boston, MA, USA), and bands were quantified using ImageJ analysis.

### 2.6. Cell Surface Biotinylation

Cell surface biotinylation was performed as previously described in order to determine CAR^Ex8^ levels at the apical surface of CAR^Ex8^ cells [17,24]. Briefly, MDCK-CAR^Ex8^ cells were polarized on 30-mm transwell inserts. PMN were added to the apical surface of MDCK-CAR^Ex8^ cells for 15 min at 37 °C as before. MDCK-CAR^Ex8^ cells were then placed on ice for 30 min and then washed three times with ice-cold PBS. Then, 1 mg/mL Sulfo-NHS-SS-biotin (ThermoFisher Scientific, Waltham, MA, USA) dissolved in PBS was added to the apical surface of cells and incubated for 1 h on ice. Cells were again washed thoroughly with ice-cold PBS and then lysed in lysis buffer {50 mM Tris pH 7.4, 150 mM NaCl, 1% Triton X-100 as well as the protease inhibitors leupeptin (10 mg/mL), apoprotinin (10 mg/mL), pepstatin (10 mg/mL), and phenyl-methylsulfonyl fluoride (1 mM) for 20 min on ice. Protein concentrations were calculated for each lysate, and volumes of lysate that contained 1 mg of protein were ultimately mixed with NeutrAvidin beads (ThermoFisher Scientific, Waltham, MA, USA) for 2 h in mini tubes spinning end-over-end at 4 °C. Beads were then washed three times with lysis buffer and ultimately placed in 2× loading dye and incubated at 65 °C for 10 min to release biotinylated proteins. These proteins were then subjected to SDS-PAGE and WB.

### 2.7. Statistical Analyses

All experiments were performed in at least triplicates at a minimum of three times. Microsoft Excel or Graph Pad Prism V5 were used to perform statistical analyses. Statistical significance was evaluated using ANOVA or *t*-test, as indicated.

## 3. Results

### 3.1. Donor Demography

Primary human neutrophils were freshly isolated 36 separate times from healthy human volunteers during the period from 2016 to 2018. The age of donors ranged from 19 to 38, with the median age being 27. There were more male (88%) than female (11%) donors. The majority of the donors were Caucasian (78%), while the rest were Asian (19%) and Mediterranean (3%).

### 3.2. Serine Protease Contributes to PMN Mediated Enhancement of Epithelial HAdV Transduction

**Figure 1:**
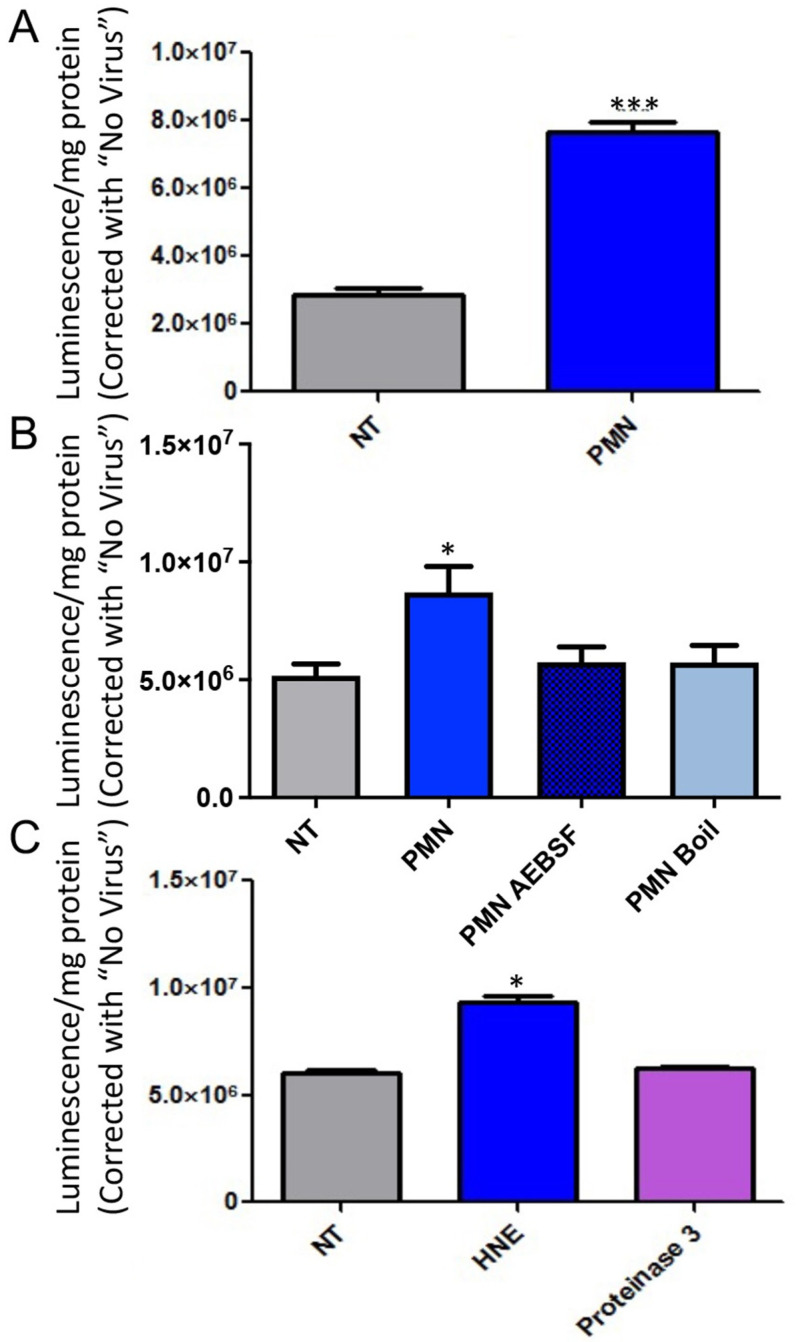
Exposure to primary human neutrophils leads to increased AdV5 transduction, inhibition of neutrophil serine proteases ablates PMN mediated enhancement of epithelial AdV5 transduction, and addition of human neutrophil elastase alone enhances AdV5 transduction of MDCK-CAR^Ex8^ epithelia. (**A**) MDCK-CAR^Ex8^ epithelia were induced with doxycycline and 24 h later exposed to PMN. Cells were infected with AdV-LacZ, and beta galactosidase assay was performed the following day to measure AdV transduction. Data are presented as means ± standard errors (SEM) from quadruplicate samples in 13 independent experiments. *** *p* < 0.001 in two tailed Students *t*-test. (**B**) Freshly isolated PMN were treated with serine protease inhibitor AEBSF or vehicle control in HBSS at 37 °C or heat killed by placing in a boiling water bath for 30 min. Confluent monolayers of MDCK-CAR^Ex8^ epithelia in 24-well plates were then exposed to these PMN before infection with AdV5-LacZ and performing beta galactosidase assay the following day. (**C**) Confluent monolayers of MDCK-CAR^Ex8^ epithelia in 24-well plates were exposed to human neutrophil elastase, neutrophil proteinase 3, or vehicle control before infection with AdV5-LacZ and subsequent beta galactosidase assay. Error bars represent SEM of 3 independent experiments, each performed in quadruplicate. * *p* < 0.05 by one-way ANOVA and Bonferroni post-hoc test. NT, non-treated; all other treatments were compared to NT.

First, we aimed to further characterize previous data that brief exposure to primary human neutrophils enhances HAdV transduction of MDCK epithelia [18]. Primary human neutrophils were isolated from the peripheral blood of healthy human donors as previously described [18] and added to a confluent monolayer of MDCK epithelial cells that stably express CAR^Ex8^ through a doxycycline inducible promoter (MDCK-CAR^Ex8^ cells) for 15 min. PMN were then washed away, and MDCK-CAR^Ex8^ cells were transduced with HAdV5 expressing a LacZ reporter gene (HAdV5-LacZ), and beta galactosidase activity (representing HAdV5 transduction) was measured 24 h post transduction. PMN exposure significantly enhanced HAdV5 transduction of the epithelia from about two-fold to seven-fold (Figure 1A) compared to non-treated cells (NT). These data confirmed our previous observation that PMN exposure consistently enhanced HAdV5 transduction among multiple PMN donors.

We then sought to determine what PMN factor(s) are responsible for enhancement of epithelial HAdV5 transduction. Neutrophil serine proteases are involved in direct microbial digestion but also facilitate cytokine signaling that can potentially affect a host’s susceptibility to viral infection [25,26]. We hypothesized that neutrophil serine protease(s) were responsible for PMN-mediated enhancement of epithelial HAdV5 transduction. To test this hypothesis, PMN were either heat killed by boiling, pretreated with the serine protease inhibitor AEBSF, or vehicle control, and then exposed to MDCK-CAR^Ex8^ cell monolayers. This was followed by subsequent inoculation with HAdV5-LacZ and measurement of HAdV5 transduction by beta galactosidase assay. Similar to heat-killed PMN, AEBSF exposure totally ablated the PMN-mediated increase in epithelial HAdV5 transduction (Figure 1B). This implicated neutrophil serine protease(s) as factor responsible for PMN-mediated enhancement of epithelial HAdV5 transduction. To determine which specific serine protease(s) may be responsible for this increase, the candidate recombinant serine proteases, human neutrophil elastase (HNE) and neutrophil proteinase 3, were tested for their ability to alter HAdV5 transduction in MDCK epithelia. Treating MDCK epithelia with 0.1 U/mL HNE resulted in a significant two-fold increase in HAdV5 transduction, a similar increase to that mediated by whole PMN. Notably, treatment with 0.1 U/mL neutrophil proteinase 3 had no significant effect on epithelial HAdV5 transduction, suggesting that only some neutrophil serine proteases are directly involved in PMN-mediated enhancement of epithelial HAdV5 transduction (Figure 1C). Taken together, our data suggest that HNE is a primary contributing factor to PMN-mediated enhancement of epithelial HAdV5 transduction.

### 3.3. PMN Exposure Does Not Increase Epithelial CAR^Ex8^ Expression

**Figure 2:**
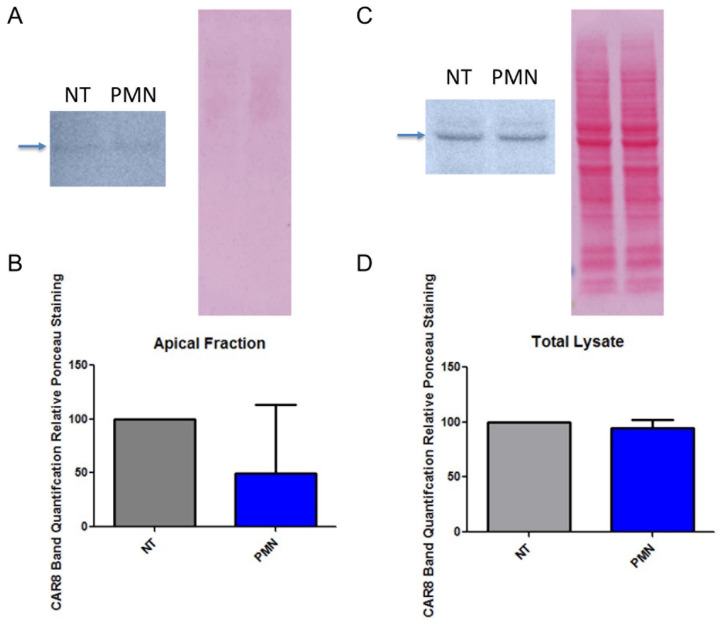
PMN exposure does not significantly change apical or total expression of CAR^Ex8^ in MDCK-CAR^Ex8^ epithelia. MDCK-CAR^Ex8^ cells were grown in 6-well transwell inserts until they were fully polarized and then exposed to PMN. Apical surface proteins were marked with sulfo-NHS-biotin and cell lysates were run through neutravidin beads to create an apical protein fraction. Cell lysates containing apical (**A**) and total (**C**) cell proteins were subjected SDS-PAGE, Ponceau staining, and subsequent Western blotting with antibody specific to CAR^Ex8^. Band quantifications are depicted for apical (**B**) and total (**D**) CAR^Ex8^ staining relative to Ponceau loading control. Error bars represent SEM from 3 independent experiments. NT, non-treated.

It has been demonstrated that interleukin 8 (IL-8) increases apical CAR^Ex8^ expression and HAdV5 transduction in epithelia [18,19]. Furthermore, evidence suggests that PMN are capable of releasing IL-8 and that HNE triggers epithelial cell lines to produce IL-8 [26,27,28]. We thus hypothesized that PMN exposure increases apical CAR^Ex8^ expression in MDCK-CAR^Ex8^ cells. Therefore, to determine the mechanism of PMN modulation of HAdV5 transduction of epithelial cell, MDCK monolayers were polarized in transwell inserts and then exposed to PMN as before. Cells were then exposed to Sulfo-NHS-biotin to mark apical proteins for proper separation from total proteins. Both protein fractions were subjected to SDS-PAGE and Western blotting. Blots were subsequently stained with Ponceau stain as a loading control and probed with an antibody specific to CAR^Ex8^. Contrary to our hypothesis, PMN exposure did not significantly change apical CAR^Ex8^ expression (Figure 2A,B) or total CAR^Ex8^ expression (Figure 2C,D) in MDCK CAR^Ex8^ cells, suggesting that the increase in epithelial HAdV5 transduction exerted by PMN through means other than the alteration of expression and/or localization of CAR^Ex8^.

### 3.4. Inhibition of Epithelial Endocytosis Does Not Decrease PMN-Mediated Enhancement of HAdV5 Transduction

**Figure 3:**
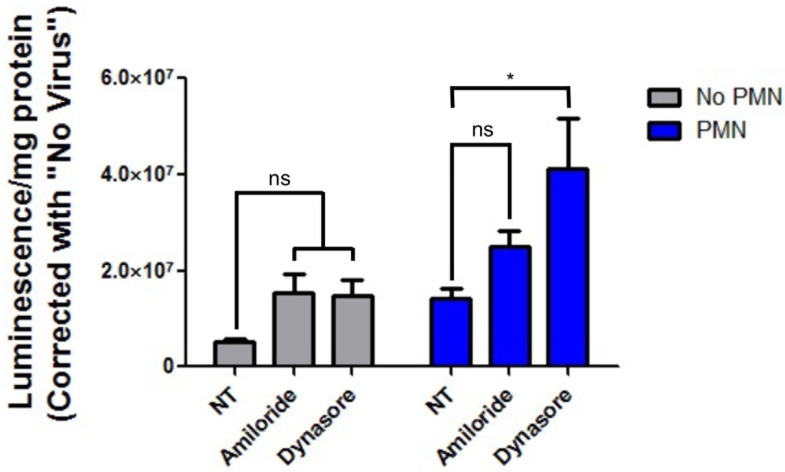
Inhibition of macropinocytosis and dynamin-dependent endocytosis pathways does not ablate PMN-mediated enhancement of epithelial AdV5 transduction. MDCK-CAR^Ex8^ cells were pretreated with amiloride or dynasore in serum-free media before exposure to PMN and subsequent infection with AdV5-LacZ, followed by beta-galactosidase activity assay the following day. Error bars represent SEM from 3 independent experiments each performed in triplicate. * *p* < 0.05 by one-way ANOVA and Bonferroni post-hoc test. NT, non-treated, ns, not significant.

Recent work has demonstrated that HAdV can enter epithelial cells through the induction of plasma membrane damage and subsequent triggering of non-specific endocytic events [29]. We then hypothesized that PMN exposure might trigger nonspecific epithelial endocytosis events that HAdV5 could co-opt for entry. If this were true, inhibitors of endocytosis should at least partially ablate the increase in HAdV5 transduction mediated by PMN. MDCK-CAR^Ex8^ cells were pretreated with inhibitors of the large GTPase dynamin (dynasore) and the Na^+^/proton exchanger necessary for macropinocytosis (amiloride) [30,31,32]. MDCK-CAR^Ex8^ cells were then subsequently exposed to PMN to determine the effect of endocytosis inhibition on PMN-mediated enhancement of epithelial HAdV5 entry. Contrary to our hypothesis, pretreatment with these inhibitors had either no effect on HAdV5 transduction (amiloride) or further increased HAdV5 transduction (dynasore), (Figure 3). This suggested that PMN-mediated triggering of epithelial macropinocytosis and/or dynamin-dependent entry did not contribute to PMN-mediated enhancement of epithelial HAdV5 transduction.

### 3.5. HNE Activates Autophagic Flux in MDCK-CAR^Ex8^ Epithelia

**Figure 4:**
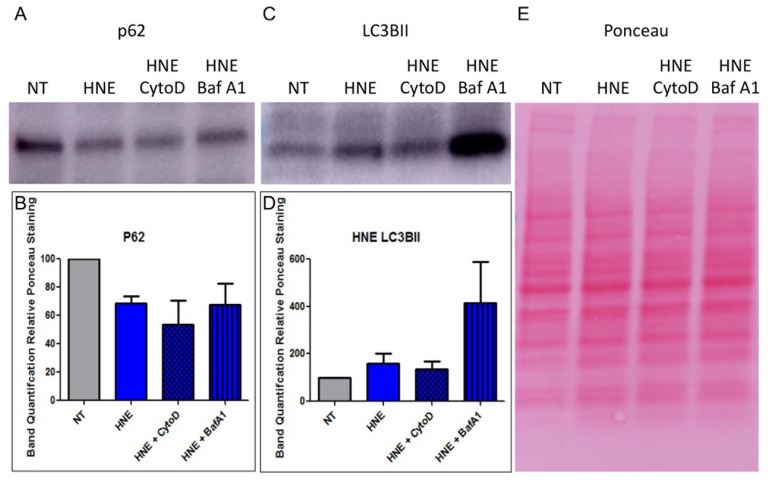
HNE treatment results in activation of autophagic flux in MDCK-CAR^Ex8^ cells. MDCK-CAR^Ex8^ cells were pretreated with cytochalasin D or bafilomycin A1 and subsequently treated with HNE. The MDCK-CAR^Ex8^ cells were then washed thoroughly before performing Western blotting on the samples. Representative Western blot data of (**A**) anti-p62-treated, (**C**) anti-LC3BII-treated blots, as well as (**E**) Ponceau staining are shown. Band quantifications of (**B**) p62 bands relative Ponceau staining and (**D**) LC3BII bands relative to Ponceau staining are shown. Error bars represent SEM from 3 independent experiments each performed in quadruplicate. NT, non-treated.

**Figure 5:**
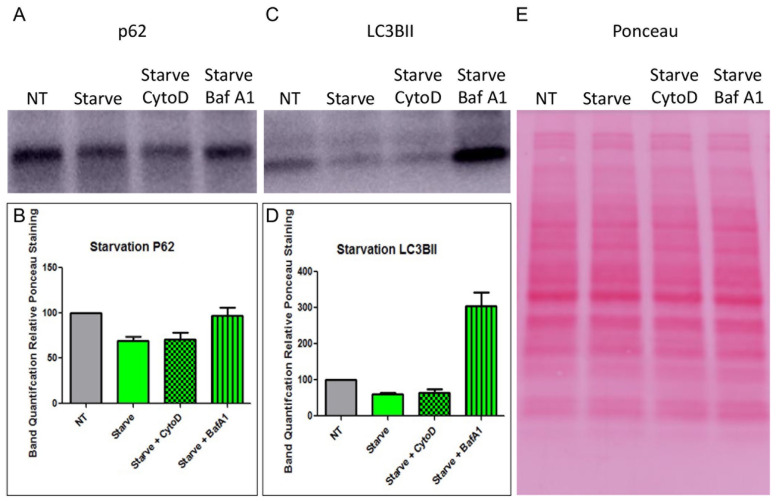
HBSS starvation results in activation of autophagic flux in MDCK-CAR^Ex8^ cells. MDCK-CAR^Ex8^ cells were starved in HBSS+/+ for 4 h and then treated with cytochalasin D or bafilomycin A1. The MDCK-CAR^Ex8^ cells were then washed thoroughly before performing Western blotting on the samples. Representative Western blot data of (**A**) anti-p62 treated and (**C**) anti-LC3BII treated blots as well as (**E**) Ponceau staining are shown. Band quantifications of (**B**) p62 bands relative Ponceau staining and (**D**) LC3BII bands relative to Ponceau staining are shown. Error bars represent SEM from 3 independent experiments each performed in quadruplicate. NT, non-treated.

It has been reported that epithelial cells that are undergoing autophagic flux are more susceptible to adenovirus transduction [14]. In addition, HNE can activate autophagic flux in airway epithelial cells [33]. We hypothesized that HNE activated autophagic flux in MDCK-CAR^Ex8^ cells, which resulted in enhanced HAdV5 transduction in these cells. To test this hypothesis, MDCK-CAR^Ex8^ cells were exposed to 0.1 U/mL HNE for 15 min and then subjected to SDS-PAGE and WB with antibodies against the important autophagic proteins p62 and LC3BII. LC3BII is a protein that is incorporated into growing autophagosomes, and its relative abundance suggests intracellular autophagosome number [34]. Alternatively, p62 is a protein that is degraded almost exclusively in autophagosomes, making its relative abundance analogous to autophagosomal degradation [34]. Thus, measuring changes in LC3BII and p62, analogous for autophagosome number and autophagosome degradation, is a well-established way of measuring autophagic flux [34]. Treatment with HNE resulted in a significant decrease in p62 staining (Figure 4A) and increase in LC3BII staining (Figure 4B) relative to vehicle-treated MDCK-CAR^Ex8^ cells. This suggested that autophagic flux had been activated by HNE treatment, resulting in increased autophagosome production and simultaneous increased rate of degradation. Autophagy inhibitors Cytochalasin D and bafilomycin A1 were used as controls in these experiments. Cytochalasin D, an F-actin inhibitor, has been demonstrated to inhibit the early stages of autophagy by inhibiting autophagosome formation, while bafilomycin A1 inhibits autophagosome-lysosome fusion [34,35]. Interestingly, in the dose and incubation time used in these experiments, we did not observe a significant change in p62 (Figure 4A) or LC3BII (Figure 4C) staining with pretreatment of Cytochalasin D. On the other hand, pretreatment with Bafilomycin A1 partially rescued p62 (Figure 4A,C), resulting in a significant increase in LC3BII staining (Figure 4B,D). To clarify that autophagic flux was driving enhancement of HAdV5 transduction in HNE-treated cells, experiments were performed in which autophagic flux was activated through a different mechanism: nutrient starvation. MDCK-CAR^Ex8^ monolayers were placed on HBSS for 4 h, lysed, and then subjected to SDS-PAGE, WB with the same anti-p62 and anti-LC3BII antibodies. Compared to control cells on complete media, nutrient starvation resulted in a decrease in p62 and LC3BII, suggesting that autophagic flux was being activated and most autophagosomes had been degraded by this time (Figure 5). Similar to HNE treated cells, pretreatment of starved cells with cytochalasin D did not significantly alter p62 (Figure 5A,B) or LC3BII (Figure 5C,D), and pretreatment with bafilomycin A1 rescued p62 staining (Figure 5A,B) and significantly increased LC3BII staining (Figure 5C,D).

### 3.6. Starvation, HNE, and Whole PMN-Mediated Enhancement of Epithelial HAdV5 Transduction in MDCK-CAR^Ex8^ Cells Is Differentially Affected by Cytochalasin D and Bafilomcyin A1

**Figure 6:**
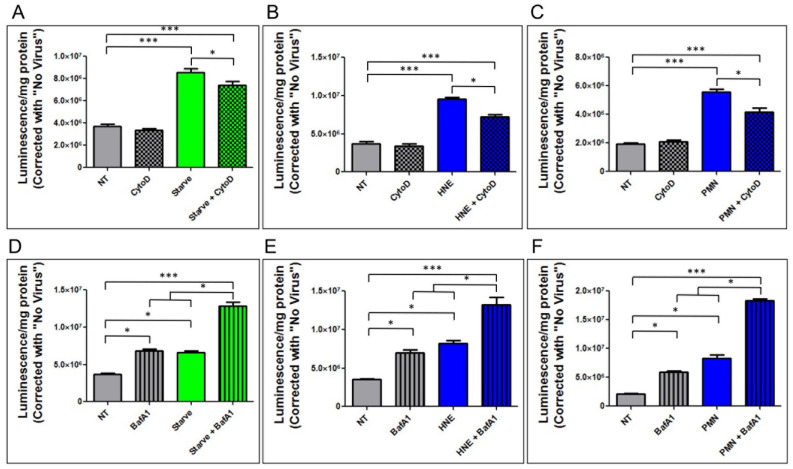
Cytochalasin D partially ablates and bafilomycin A1 further exacerbates enhancement of epithelial AdV5 transduction. MDCK-CAR^Ex8^ cells were either (**A**) exposed to HBSS starvation and then treated with cytochalasin D before treatment with (**B**) HNE or (**C**) PMN. MDCK-CAR^Ex8^ cells were then either (**D**) exposed to HBSS starvation and concurrently treated with bafilomycin A1 or treated with bafilomycin A1 and then subsequently treated with (**E**) HNE or (**F**) PMN. In all cases, the MDCK-CAR^Ex8^ cells were subsequently infected with AdV5-LacZ. Beta-galactosidase activity assay was performed the following day to measure AdV5 transduction. Error bars represent SEM from 3 independent experiments each performed in quadruplicate. * *p* < 0.05, *** *p* < 0.001 by one-way ANOVA and Bonferroni post-hoc test. NT, non-treated.

Given the previous data, we then set out to determine if activation of autophagic flux in MDCK-CAR^Ex8^ cells with HBSS starvation would result in a similar enhancement of HAdV5 transduction compared to HNE and whole PMN. MDCK-CAR^Ex8^ cells were either subjected to nutrient starvation in HBSS for 4 h, treatment with 0.1 U/mL HNE, or treatment with 2 × 10^6^ PMN in the presence and absence of cytochalasin D or bafilomycin A1 pretreatment. Cells were then washed and inoculated with HAdV5-LacZ and adenoviral transduction was measured via beta-galactosidase assay. Interestingly, pretreatment with autophagic flux inhibitors cytochalasin D and bafilomycin A1 had differing results on epithelial HAdV5 transduction. While cytochalasin D treatment alone had no effect on HAdV5 transduction, pretreatment with cytochalasin D partially ablated starvation- (Figure 6A), HNE- (Figure 6B), and PMN (Figure 6C)-mediated enhancement of HAdV5 transduction. By contrast, treatment with bafilomycin A1 alone significantly increased HAdV5 transduction and further exacerbated the starvation- (Figure 6D), HNE- (Figure 6E), and PMN (Figure 6F)-mediated enhancement of epithelial HAdV5 transduction.

## 4. Discussion

Viruses are in a constant arms race against host immune defenses in order to establish infections and replicate their genomes. Neutrophils employ a variety of molecular strategies that can impede microbial infections [36]. For example, airway epithelial cells are less likely to be infected by respiratory syncytial virus when they have been co-cultured with primary human PMN, likely due to PMN degranulation [37]. HAdV2 has been shown to be effectively phagocytized and destroyed by primary human PMN in the presence of preformed opsonizing antibodies [38]. In contrast, our data demonstrate that primary human PMN reliably enhance HAdV5 transduction of an MDCK epithelial model system. There is variability in the degree to which primary human PMN enhance epithelial HAdV5 transduction, which is likely to due to a variety of PMN biological factors that differed between donors. In this manuscript, we present data suggesting that PMN serine protease-mediated activation of epithelial autophagy partially explains this phenomenon. However, it is important to note that additional undiscovered neutrophil and epithelial factors may also contribute.

The primary receptor for most human adenoviruses, CAR, functions not only as a homophilic transmembrane protein at epithelial junctions but also binds neutrophilic JAM-L and aids in PMN transepithelial migration [20,21]. The apical isoform of CAR, CAR^Ex8^, can anchor PMN at the epithelial surface, and its expression can be upregulated by IL-8 signaling, which predisposes epithelial cells to HAdV5 infection from the apical surface [18,19]. We were surprised to find that PMN, which themselves produce IL-8, did not enhance epithelial CAR^Ex8^ expression. In fact, our data trended towards a decrease in CAR^Ex8^ expression in the presence of PMN, suggesting that PMN proteases may be degrading apical proteins. This is consistent with a report that PMN presence at the apical surface results in some degree of apical ZO-1 degradation although this report used a higher PMN-epithelial cell ratio than in our study [37]. Furthermore, we demonstrate that pretreatment with amiloride and dynasore do not ablate PMN-mediated enhancement of HAdV5 transduction, suggesting that neither epithelial macropinocytosis nor epithelial dynamin-dependent entry mechanisms are involved. It is unclear why dynasore pretreatment increased HAdV5 transduction in PMN-treated MDCK-CAR^Ex8^ cells. However, it is possible that dynasore treatment of these cells further exacerbated autophagic flux, as has been reported to happen in HEK-293 cells [39].

Our data demonstrate that PMN serine proteases are responsible for PMN-mediated enhancement of HAdV5 transduction, as inhibition of PMN serine proteases with AEBSF completely ablated this effect. Furthermore, we demonstrate that treatment with HNE recapitulates the increase in HAdV5 transduction of MDCK-CAR^Ex8^ cells mediated by exposure to PMN. Serine proteases have been implicated in a variety of biological processes, including direct pathogen degradation, leukocyte-epithelial signaling, and facilitation of inflammatory cascades [40,41,42]. Furthermore, serine proteases, and especially HNE, have been implicated as significant contributors to host tissue destruction in severe influenza and COVID-19 infections, leading some to suggest that elastase inhibitors may have clinical utility in severe COVID-19 pneumonia [43,44]. Our data suggest the possibility that the use of elastase inhibitors may exhibit some antiviral effects in addition to sparing organ systems from inflammatory damage. While it certainly stands to reason that HNE increasing epithelial HAdV5 transduction would also likely increase the virus’s replicative capacity and pathogenesis, further experiments in animal models would be required to test if this increase in epithelial transduction correlates to clinical infection. While it has been demonstrated that HNE can activate autophagic flux in airway epithelial cells through the activation of MAPK8/JNK 1 and MAPK14/p38aplha MAPK pathways, the molecular mechanism governing HNE-mediated activation in autophagic flux in MDCK-CAR^Ex8^ cells is unknown [33]. Future experiments will attempt to identify the target(s) responsible.

Our data also demonstrate that activation of autophagic flux, either with nutrient starvation or HNE exposure, enhances HAdV5 transduction in our MDCK-CAR^Ex8^ cells. Furthermore, we show that this effect is partially ablated with brief epithelial pretreatment with F-actin inhibitor Cytochalasin D. Interestingly, Cytochalasin D pretreatment did not significantly affect levels of LC3BII or p62 and is therefore not significantly altering epithelial autophagic flux in our experiments. Future studies will investigate whether F-actin inhibition with cytochalasin D partially ablates autophagy-mediated enhancement in epithelial HAdV5 infection through direct inhibition of autophagy vs. through other related cellular processes, such as endosomal trafficking. Importantly, treatment with cytochalasin D alone did not inhibit HAdV5 transduction of MDCK-CAR^Ex8^ cells. Longer treatments with cytochalasin D (90 min) have been demonstrated to inhibit HAdV entry in some cell types; however, in our experiments, cells are treated for significantly less time (20 min), and cytochalasin D is washed away before HAdV5 is added to the cells [45]. Through an unknown mechanism, bafilomycin A1 treatment increases HAdV5 transduction in MDCK-CAR^Ex8^ cells and further exacerbates autophagy-mediated enhancement of HAdV5 transduction. This could be explained by multiple possible mechanisms, including rescuing of some portion of HAdV5 virions from lysosomal degradation or through the relative enrichment of an endosomal population that HAdV5 can more efficiently escape from. Additional experiments beyond the scope of the current work will be required to further elucidate the molecular mechanisms that govern autophagy-mediated enhancement of HAdV5 transduction. Furthermore, the possibility that differences in the early stages of infection exist between MDCK and human airway epithelial cells cannot be discounted. Future experiments are planned to validate the findings discussed in this manuscript in primary human airway epithelia. 

HAdV is a pathogenic virus that can cause a wide variety of severe infections, especially in immunocompromised patients [2]. Furthermore, HAdV has been used as a reliable vector delivery system not only in the research setting but also as effective vaccines to fight the COVID-19 pandemic and various cancers [6,7,8]. In this manuscript, we show that PMN serine proteases enhance HAdV5 transduction of an epithelial model system without affecting levels of CAR^Ex8^. We then demonstrate that exposure to HNE alone is sufficient to recapitulate this effect and that HNE treatment activates epithelial autophagic flux. We also demonstrate that F-actin inhibition partially reverses the increase in HAdV5 transduction mediated by autophagic flux, while inhibiting autophagosome-lysosome fusion exacerbates it.

**Figure 7:**
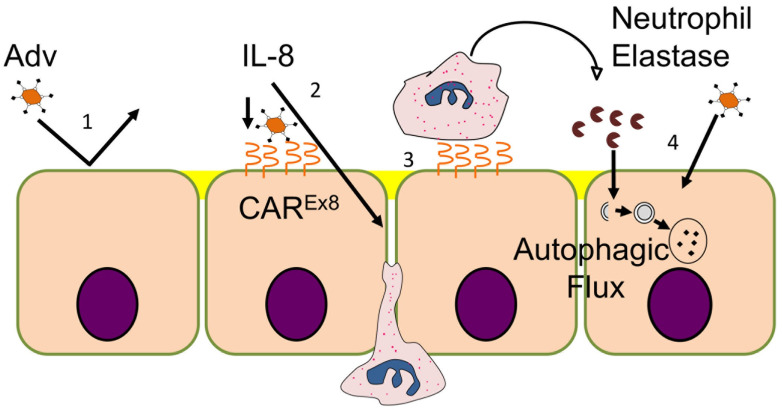
Summary model of how innate immune factors regulate epithelial susceptibility to AdV5 infection. (1) Under normal circumstances, polarized epithelia express very low levels of apical CAR^Ex8^, making them relatively resistant to apical AdV infection. (2) AdV-5 triggers release of proinflammatory cytokine IL-8, which induces apical expression of CAR^Ex8^ and (3) transepithelial migration of neutrophils. (4) Once neutrophils arrive, release of HNE further enhances AdV-5 transduction of epithelia through a process that involves activation of epithelial autophagic flux.

In contrast to conventional wisdom that PMN exposure inhibits virus infections, our data indicate that PMN inflammation enhances HAdV transduction into epithelial cells. This leads to the overarching conclusion that HAdV5 triggers the release of multiple pro-inflammatory cytokines, including IL-8, by both epithelial cells and leukocytes [19]. These cytokines enhance apical CAR^Ex8^ expression in polarized epithelia, which results in enhancement of epithelial HAdV5 transduction [18]. Here, we demonstrate that PMN, and specifically neutrophil serine protease HNE, further enhance HAdV5 transduction through a process that involves activation of autophagic flux (Figure 7).

## Data Availability

The data presented in this study are available upon request from the corresponding author. The data are not publically available to ensure the privacy of human research subjects.
